# Bacterial and Fungal Co-Infections and Superinfections in a Cohort of COVID-19 Patients: Real-Life Data from an Italian Third Level Hospital

**DOI:** 10.3390/idr14030041

**Published:** 2022-05-12

**Authors:** Manuela Ceccarelli, Andrea Marino, Sarah Pulvirenti, Viviana Coco, Barbara Busà, Giuseppe Nunnari, Bruno Santi Cacopardo

**Affiliations:** 1 Department of Clinical and Experimental Medicine, Unit of Infectious Diseases, University of Catania, I-95122 Catania, Italy; manuela.ceccarelli@unict.it; 2 Department of Biomedical, Dental, Morphological and Functional Imaging Sciences, Unit of Infectious Diseases, University of Messina, I-98124 Messina, Italy; 3 Unit of Infectious Diseases, ARNAS “Garibaldi”, “Nesima” Hospital, I-95122 Catania, Italy; andreamarino9103@gmail.com (A.M.); sarah.pulvirenti@libero.it (S.P.); 4 Department of Biomedical and Biotechnological Sciences, Unit of Infectious Diseases, University of Catania, I-95123 Catania, Italy; 5 Department of Clinical and Experimental Medicine, Unit of Infectious Diseases, University of Messina, I-98124 Messina, Italy; giuseppe.nunnari@unime.it; 6 Unit of Hospital Pharmacy, ARNAS “Garibaldi”, “Garibaldi” Hospital, I-95124 Catania, Italy; vivicoco@hotmail.it (V.C.); b.busa@ao-garibaldi.ct.it (B.B.)

**Keywords:** superinfections, coinfections, bacterial, fungal, COVID-19, hospital-acquired infection (HAI), community-acquired infection (CAI)

## Abstract

The use of immune suppressive drugs combined with the natural immune suppression caused by SARS-CoV-2 can lead to a surge of secondary bacterial and fungal infections. The aim of this study was to estimate the incidence of superinfections in hospitalized subjects with COVID-19. We carried out an observational retrospective single center cohort study. We enrolled patients admitted at the “Garibaldi” hospital for ≥72 h, with a confirmed diagnosis of COVID-19. All patients were routinely investigated for bacterial, viral, and fungal pathogens. A total of 589 adults with COVID-19 were included. A total of 88 infections were documented in different sites among 74 patients (12.6%). As for the etiology, 84 isolates were bacterial (95.5%), while only 4 were fungal (4.5%). A total of 51 episodes of hospital-acquired infections (HAI) were found in 43 patients, with a bacterial etiology in 47 cases (92.2%). Community-acquired infections (CAIs) are more frequently caused by *Streptococcus pneumoniae*, while HAIs are mostly associated with *Pseudomonas aeruginosa*. A high rate of CAIs and HAIs due to the use of high-dose corticosteroids and long hospital stays can be suspected. COVID-19 patients should be routinely evaluated for infection and colonization. More data about antimicrobial resistance and its correlation with antibiotic misuse in COVID-19 patients are required.

## 1. Introduction

The pandemic caused by the Severe Acute Respiratory Syndrome Coronavirus-2 (SARS-CoV-2), etiologic agent of the Coronavirus Infectious Disease COVID-19, has exhausted health care systems worldwide; many countries are still dealing with outbreaks of COVID-19 and its complications [1,2,3]. In addition to severe respiratory symptoms, the virus causes an aberrant activation of the immune system, with an increased production of inflammatory cytokines and a consequent immune suppression [4,5,6,7,8].

To fight the inflammatory syndrome, many immunomodulant agents are used, such as corticosteroids, anti-interleukin-1 (IL-1), anti-interleukin-6 (IL-6), and inhibitors of the JAK-STAT pathway [9,10,11,12,13,14,15,16,17,18]. The use of immune suppressive drugs, combined with the natural immune suppression caused by the virus, can lead to a surge of secondary bacterial and fungal infections [8,19,20,21,22,23,24,25,26,27,28,29,30,31,32,33,34,35].

During COVID-19 patients’ hospital admission, biologic specimens, such as blood and sputum, are repeatedly collected and sent for cultures to ascertain the presence of bacterial or fungal co-infections and superinfections [21,36]. However, the prevalence of bacterial and fungal co-infections and superinfections is still controversial [21].

With this study, we aimed to estimate the incidence of superinfections in hospitalized subjects with COVID-19, by discriminating between the origins of community-acquired infections (CAIs) from hospital-acquired infections (HAIs); secondly, we assessed the different impacts on length of hospital stay and mortality.

## 2. Materials and Methods

### 2.1. Study Design

This observational retrospective cohort study was performed at the “Garibaldi” Hospital in Catania, a 700-bed University Center that provides broad and specialized medical, surgical, and intensive care to an urban area populated by more than 500,000 adult individuals.

### 2.2. Inclusion Criteria

All patients admitted at the “Garibaldi” Hospital for ≥72 h between 1 October 2020, and 28 February 2021, were enrolled in this study if they had a COVID-19 diagnosis confirmed by real-time reverse transcription polymerase chain reaction (RT-PCR) testing performed on nasopharyngeal swab specimens.

### 2.3. Data Collection

Sensitive data of patients (name, surname, date of birth, attribution of study ID) were collected on paper and saved separately from other data. They were only accessible to the PI, Co-PI, and the center manager. All the data about the patients included in this study were anonymously collected in an electronic database only accessible to the researchers. We collected epidemiologic data (study ID, sex, age, date of the onset of symptoms, date of the diagnosis, date of admission); past clinical history (hypertension, chronic heart disorders, diabetes, chronic obstructive pulmonary disease (COPD), chronic kidney failure (CKF), obesity, other); outcome (date, outcome); microbiological data (material, date of culture, isolate); infectious diseases specialist consultation (date).

At hospital admission all patients were routinely investigated for bacterial, viral, and fungal pathogens in blood, in normally sterile fluid, such as urine and cerebral-spinal fluid (CSF), and in sputum, with standard microbiological procedures (cultures, PCR). A urinary antigenic test was performed in all cases for *Legionella* spp. and *Streptococcus pneumoniae* infection.

Bacterial respiratory superinfection was confirmed in patients with one or more positive cultures for respiratory pathogens obtained from blood, pleural fluids, good-quality sputum and bronchoalveolar lavage, and/or a positive urinary antigen test. Good-quality sputum was defined by the presence of at least 25 polymorphonuclear leukocytes (PMNLs).

Bacteria and fungi were identified in positive cultures with Vitek^®^ 2 (Biomérieux, Craponne, France), an automated system which also allows to perform antimicrobial susceptibility tests (AST) with a card by the same producer. AST cards are chosen among those available, specific for *Enterobacteriaceae*, non-fermenters, *Staphylococci*, *Enterococci*, *Streptococci* and *Yeasts*, after identification.

Bloodstream infections (BSIs) were defined as the growth of a non-skin flora commensal on one or more blood culture.

Urinary tract infection (UTI) was defined as the growth of bacteria or fungi in a cultured urine sample from a patient with either urinary clinical symptoms or radiologic evidence, by ultrasounds (US), computerized tomography (CT) scan, or magnetic resonance imaging (MRI), of urogenital disease.

All these infections were categorized as:

1. CAIs, if the diagnosis was made at the time or within the first 48 h of hospital admission
2. HAIs, if the diagnosis was made >48 h after the hospital admission.

The hospital pharmacy gave us the information about antibiotic use in COVID-19 wards. We analyzed data about total use and compared the antibiotic prescription of those only prescribed drugs by the infectious disease consultant as part of the antimicrobial stewardship with pre-pandemic use. We calculated the total amount of drug used in grams (g), then divided per the defined daily dose (DDD) taken from the ATC/DDD index 2022 [37].

### 2.4. Statistical Analysis

Data were collected anonymously in an electronic data sheet (Microsoft Excel for Mac, version 16.37, Microsoft, Redmond, Washington, DC, USA).

Statistical analysis was carried out with GraphPad Prism version 9.2.0 for macOS (GraphPad Software, San Diego, CA, USA). Categorical variables were analyzed with descriptive (count, percentage) and inferential (chi-square test) statistics. Quantitative variables were analyzed with descriptive (median and interquartile range, IQR) and inferential statistics. The distribution of the quantitative variables was deemed as normal or non-normal using Kolmogorov–Smirnov test. Normally distributed variables were tested with parametric tests such as *t*-test and ANOVA. Non-normally distributed variables were tested with non-parametric tests, such as Mann–Whitney and Kruskal–Wallis. Post-hoc comparisons were carried out with Tukey’s multiple comparisons test for normally distributed variables and with Dunn’s multiple comparisons test for non-normally distributed variables. Statistical significance was set with a *p* value < 0.05.

### 2.5. Ethical Approval and Informed Consent

This research was conducted according to the Declaration of Helsinki. It was approved as a retrospective minimally invasive experimental study by the Provincial Review Board of Messina on 29 June 2020, with the protocol number 63/20 bis. Patients signed written informed consent for the use of their data for research purposes at the admission.

## 3. Results

A total of 589 adults with COVID-19 were admitted at our hospital during the study period. Of these, 395 (67.1%) were male; the median age of the population was 59 years (IQR 43–73), although the male population was slightly older (median 63 years, IQR 50–74) than the female one (median 60 years, IQR 50–73.5). Table 1 summarizes the characteristics of the patients admitted with COVID-19.

**Figure 1:**
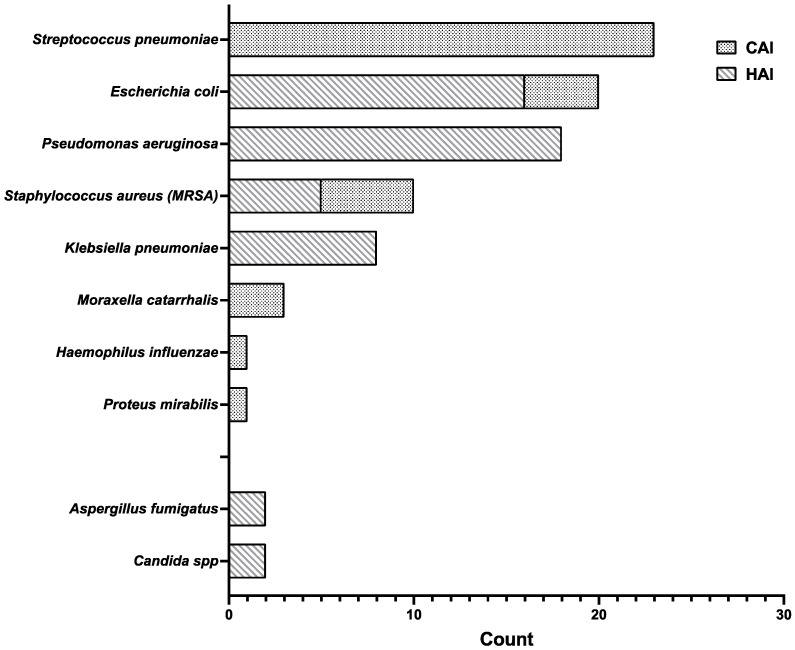
Etiology of bacterial and fungal infections, differentiated by their origin. Community-acquired infections (points) are only bacterial in etiology, while hospital-acquired infections (stripes), more frequent than CAIs, are both bacterial and fungal. Abbreviations: MRSA, methicillin-resistant *Staphylococcus aureus*; CAI, community-acquired infection; HAI, hospital-acquired infection.

A total of 88 infections were documented in different sites among 74 patients (12.6%). Mostly, male patients were affected by co-infections (45; 60.8%). As for the etiology, 84 isolates were bacterial (95.5%), while only 4 were fungal (4.5%). Figure 1 details the etiology of both bacterial and fungal infections and their distribution in CAIs and HAIs. No difference was found between male patients and female patients regarding the number of co-infections (*p* = 0.1807).

### 3.1. Community Acquired Infections

**Figure 2:**
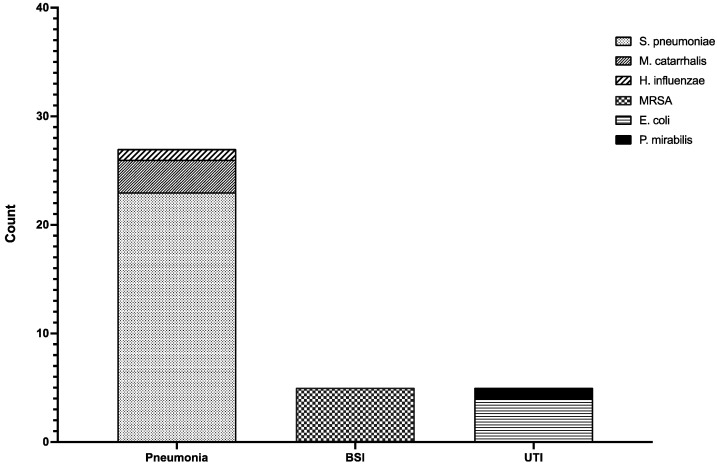
Etiology of CAIs by site of infection. Twenty-three cases of pneumonia were caused by *Streptococcus pneumoniae* (sparse points), three cases were due to *Moraxella catarrhalis* (thick points), and one patient had a *Haemophilus influenzae* (slant stripes) pneumonia. All the BSIs were caused by MRSA (checks). UTIs were caused by *Escherichia coli* (horizontal stripes) in four cases and *Proteus mirabilis* (black) in one case. Abbreviations: MRSA, methicillin-resistant *S. aureus*; BSI, bloodstream infection; UTI, urinary tract infection.

Figure 2 shows etiologies by site of infection.

Bacterial pneumonia was documented in 27 patients contemporary to COVID-19 diagnosis. Diagnosis of community-acquired bacterial pneumonia was performed with *S. pneumoniae* urinary antigen test in 17 cases. Good-quality sputum tested positive for *Streptococcus pneumoniae* in 12 cases (four of which were not identified by urinary antigen testing) and *Moraxella catarrhalis* in three cases. Bronchoalveolar lavage (BAL) tested positive *for S. pneumoniae* in one case, *Haemophilus influenzae* in one case and *M. catarrhalis* in three cases, all of which were also identified by sputum culture.

In five cases, a diagnosis of community-acquired BSI was made. All of them were caused by methicillin-resistant *Staphylococcus aureus* (MRSA) found in blood cultures taken upon the admission.

Finally, five UTIs were identified: three cystitis and two acute pyelonephritis. Two of the cases of cystitis were caused by *Escherichia coli*, while one was caused by *Proteus mirabilis*. All the acute pyelonephritis cases in this cohort were caused by *P. mirabilis*. All five patients with UTIs had undergone urinary catheterization before the onset of COVID-19.

### 3.2. Hospital Acquired Infections

A total of 51 episodes of HAI were found in 43 patients, with a bacterial etiology in 47 cases (92.2%). The most common HAI was pneumonia, diagnosed in 40 patients. Of these infections, 25 episodes occurred in patients admitted at the intensive care unit (ICU).

**Figure 3:**
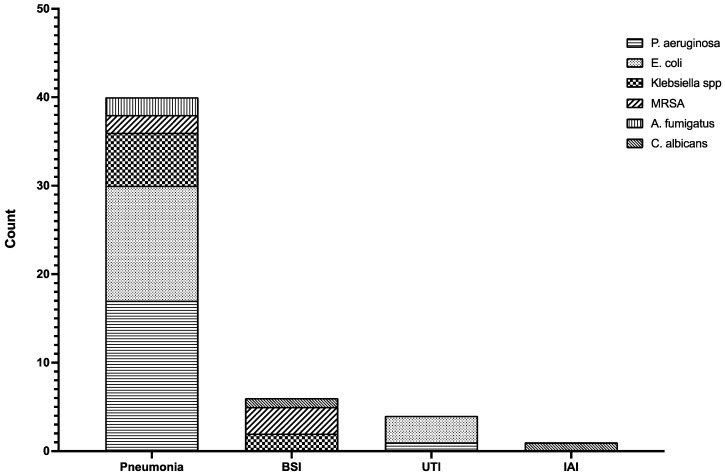
Etiology of HAIs by site of infection. A total of 18 cases of pneumonia were caused by *Pseudomonas aeruginosa* (horizontal stripes), 13 cases were due to *Escherichia coli* (sparse points), 6 patients had a *Klebsiella* spp. (checks) pneumonia, and MRSA (large slant stripes) and *Aspergillus fumigatus* (vertical stripes) caused 2 cases of pneumonia each. BSIs were caused by *Klebsiella* spp. (checks) in two cases, MRSA (slant stripes) in three cases, and by *Candida albicans* (close slant stripes). UTIs were caused by *Escherichia coli* in three cases and *Pseudomonas aeruginosa* in one case. One case of IAI was caused by *C. albicans*. Abbreviations: spp., species plures; MRSA, methicillin-resistant *S. aureus*; BSI, bloodstream infection; UTI, urinary tract infection; IAI, intra-abdominal infections.

Figure 3 shows the etiologies by site of infection.

Median time from hospital admission to superinfection diagnosis was 10.6 days (IQR 5–12.8).

Multidrug resistant (MDR) microorganisms were identified in nine cases (17.6%): four cases of MDR *P. aeruginosa*, three cases of extended-spectrum beta-lactamase (ESBL) producer *E. coli*, two cases of ESBL producer *K. pneumoniae*. All the isolated *Staphylococcus aureus* were methicillin-resistant (MRSA). Table 2 shows details about the resistance panels of these MDR micro-organisms.

Four patients were affected by fungal superinfection. Two of them had lower respiratory infections caused by *Aspergillus fumigatus*. Both the patients were also affected by previous chronic lung diseases and were older than 70 years. These patients were admitted at the ICU and underwent mechanical ventilation support. They had been also treated with high-dose corticosteroids. One patient had *Candida albicans* BSI, while one, who had previously undergone abdominal surgery to remove colonic cancer, was affected by *C. albicans* peritoneal infection with ascites. All those patients affected by fungal superinfections died.

### 3.3. Outcomes

Overall mortality in our population was 10.0% (59/589).

A total of 44 patients (44/74, 59.5%) with superinfections were admitted in an ICU, 19 (25.7%) were diagnosed with a CAI, while 25 (33.8%) with a HAI.

Comprehensively, 13 patients (17.6%) diagnosed with a superinfection died: nevertheless, by stratifying mortality for patients with CAI and HAI a difference could be highlighted. In fact, only 3 patients (9.7%) diagnosed with a CAI died, whereas 10 patients (23.3%) with a HAI died. This difference was not statistically significant (*p* = 0.215).

Moreover, patients with superinfections stayed in the hospital for a higher number of days (median 19.2 days, IQR 12.6–25) compared to patients without bacterial or fungal infection (median 12.4 days, IQR 7.5–15) (*p* < 0.001).

### 3.4. Antibiotic Use

The hospital pharmacy gave us the data about total use of antibiotics in COVID-19 wards in 2020. We analyzed the total use in days of treatment, by dividing the total use in grams per the DDD.

We found out that in 2020 the total number of days of all antibiotic treatments in COVID-19 wards was 57,537. The six most used antibiotics were piperacillin/tazobactam (7221 days), ceftriaxone (6639 days), levofloxacin (6125 days), trimethoprim/sulfamethoxazole (5408 days), clarithromycin (4669 days), and azithromycin (4185 days). It is interesting to notice that none of these six antibiotics belong to the restricted list of antibiotics that need an infectious disease specialist authorization.

**Figure 4:**
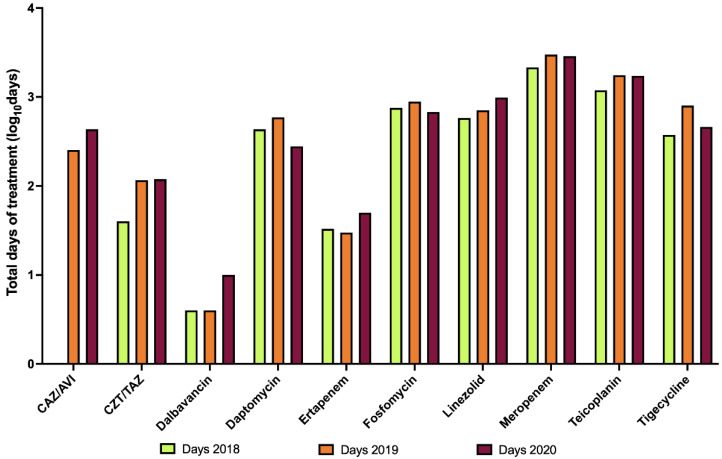
Total use of antibiotics belonging to a restricted list in COVID-19 wards, during the first year of the pandemic (Days 2020) compared with total use within the same wards during the two pre-pandemic years (days 2018, days 2019). Y axis is in logarithmic scale. It can be noticed that in 2020 the use of each antibiotic was constant or even lower than the years before, despite an increase in beds, apart from dalbavancin and ertapenem. Both antibiotics are easily managed in a day hospital setting, as they have, respectively, weekly and daily intake.

In fact, only considering the antibiotics within the list, we found a decreased overall number of days in 2020 compared to 2019. It was surprising, since during conversion to COVID-19 wards a higher number of patients were admitted, compared with normal activity periods. However, the use of single antibiotics was constant or even lower than the previous years (Figure 4).

## 4. Discussion

Bacterial superinfections during viral illnesses are usually associated with poor outcomes [21,38]. In our cohort of 589 patients affected by COVID-19 pneumonia throughout a 5-month period, we showed a 10% mortality rate, a percentage similar to the overall mortality in Italy, as reported by the Istituto Superiore di Sanità (Italian National Institute of Health–ISS) in the same period.

Some studies have been published on the incidence and prevalence of bacterial and fungal superinfections in patients affected by COVID-19 pneumonia, showing similar results. Scott et al. [21] compared a population of COVID-19 patients admitted during 2020 with patients affected by non-COVID-19 pneumonia admitted during 2019. Scott et al. showed that patients affected by COVID-19 pneumonia were less likely to have a bacterial respiratory superinfection. However, they were burdened by a higher mortality rate [21]. Moreover, they showed that the most commonly isolated micro-organism in COVID-19 patients and non-COVID-19 patients was *Staphylococcus aureus* [21].

Differently from Scott et al., we stratified the patients according to the onset of the bacterial infection in community-acquired (CA) co-infections and HAI superinfections [21]. In COVID-19 patients affected by CAP the most frequent etiologic agent was *Streptococcus pneumoniae*. Among those affected by HAP *Pseudomonas aeruginosa* was found to be the most common cause instead.

This difference from the Scott et al. data could be linked to the local epidemiology of respiratory bacterial infections. However, Scott et al. did not present any data about their local epidemiology, therefore this hypothesis cannot be either confirmed or rejected. Moreover, the higher rate of *P. aeruginosa* infections in our experience could also be related to the longer hospital stay in our cohort [21]. As a matter of fact, *Pseudomonas aeruginosa* is the most common environmental microbe in our hospital after *Acinetobacter baumannii*, and it is known that a long hospital stay increases the risk of being colonized and of developing an infection. Moreover, only considering COVID-19 wards, *Pseudomonas* spp. are the most common microbes (data not shown).

Garcia-Vidal et al. [39] reported results comparable to ours. They show that in a population of 989 consecutive patients admitted with COVID-19, the incidence of CA co-infections was 3.1%, with *Streptococcus pneumoniae* and *Staphylococcus aureus* being the most frequent isolated pathogens. Moreover, similarly to us, they showed that most of the hospital-acquired (HA) superinfections were caused by *Pseudomonas aeruginosa* and *Escherichia coli* [39]. On the contrary, Cohen et al. [22] showed a higher rate (60%) of CA co-infections diagnosed upon admission with the help of molecular tests. In particular, they highlighted a high prevalence of *Haemophilus influenzae* co-infection (36%) and methicillin-sensitive *Staphylococcus aureus* (MSSA).

Hughes et al. [26] highlighted a high prescription rate of antibiotics in patients with COVID-19 pneumonia, with special reference to antibiotics against Gram-negative microorganisms. Our antibiotic use data confirm their observation. In fact, in our COVID-19 wards, the most used antibiotic was piperacillin/tazobactam, a beta-lactam/beta-lactamase inhibitor combination which use is highly recommended in community bacterial pneumonitis needing admission in hospital, but also in UTI, abdominal infections, and infections caused by sensitive Gram-negative bacteria. Hughes also studied an approach based on the use of procalcitonin (PCT) to stratify people at risk of having a bacterial co-infection or superinfection within the first 72 h from admission in order to reduce the improper use of empiric antibiotics [26].

Temperoni et al. [27] showed a 64.5% rate of MDR microorganisms in patients admitted in the ICU setting. Our data enormously differ from theirs, as we only highlighted nine MDR Gram-negative pathogens over 88 infectious events (10.2%). However, this difference is justified by the fact that Temperoni’s study only reports data regarding 89 ICU patients, while in our case we also included patients from other wards [27].

Cona et al. [28] studied incidence, risk factors and outcome of patients with BSI and COVID-19, showing that the most common cause of BSI in a cohort of COVID-19 patients was *Staphylococcus aureus* and that CA-BSI were as frequent as HA-BSI32.

They also reported that the presence of BSI increased the length of the hospital stay [28]. Similarly, our data showed that any kind of bacterial superinfection, with particular reference to HAIs, significantly increased the length of hospital stay.

Finally, Bartoletti et al. [19] showed a high rate of invasive pulmonary aspergillosis in patients with COVID-19 who underwent mechanical ventilation. Our rate is lower, but we did not routinely look for bronchoalveolar lavage galactomannan in patients undergoing mechanical ventilation, therefore our data could have underestimated the rate of pulmonary fungal infections.

Despite some limitations, such as no data from non-COVID-19 cohorts and no data regarding antibiotic treatments, our study highlights a relevant warning. COVID-19 patients are burdened by a high rate of bacterial and fungal co-infections and superinfections. Co-infections and superinfections worsen patients’ prognosis and increase the mortality rate. Therefore, they should be routinely looked for upon admission and in each patient suffering from a worsening of the radiologic and clinical picture despite high-dose corticosteroids.

## 5. Conclusions

Our data show a rate higher than 10% of bacterial and fungal co-infections and superinfections. CAIs are more frequently caused by *Streptococcus pneumoniae*, while HAIs are mostly associated with *Pseudomonas aeruginosa*. The availability of studies describing incidence, prevalence and etiologies of CAIs and HAIs in COVID-19 patients is limited. A high rate of CAIs and HAIs due to the use of high-dose corticosteroids and long hospital staying can be suspected. More data about antimicrobial resistance and whether it is correlated with antibiotic misuse in COVID-19 patients are required. Further studies are necessary to ascertain the incidence and prevalence of co- and super-infections.

## Figures and Tables

**Table 1:** Baseline characteristics of the patients admitted at the “Garibaldi” Hospital in Catania during the period October 2020–February 2021 with COVID-19.

Sex	Male	395	67.1%
	Female	194	32.9%
Median Age (IQR), years		59 (43–73)	
Comorbidities	Hypertension	286	48.6%
	CVD	109	18.5%
	COPD	37	6.3%
	Obesity	97	16.5%
	CKD	74	12.6%
	Diabetes	127	21.6%
Vital signs	Fever	407	69.1%
	Tachypnea	180	30.6%
	SpO_2_ ≥ 94%	406	68.3%
	SpO_2_ 89–93%	74	12.6%
	SpO_2_ < 89%	109	18.5%
Laboratory tests	Leukopenia (WBC < 4000 cells/µL)	74	12.6%
	Lymphocytopenia (<1000 cells/µL)	256	43.5%
	D-dimer > 750 mg/L	273	46.3%
	CRP > 5 mg/dL	187	31.7%
	PCT > 0.5 ng/mL	28	4.7%
	AST > 40 UI/L	178	30.2%

Abbreviations: IQR, interquartile range; CVD, cardiovascular disease; COPD, chronic obstructive pulmonary disease, CKD, chronic kidney disease, SpO_2_, oxygen saturation; WBC, white blood cells; CRP, C reactive protein; PCT, procalcitonin; AST, aspartate aminotransferase.

**Table 2:** Susceptibility to antibiotic classes for the micro-organisms classified as multidrug resistant.

Microorganism	Penicillins	Cephalosporins	Carbapenems	Aminoglycosides	Fluoroquinolones	Fosfomycin	Sulfonamides	Tigecycline	Colistin
*P. aeruginosa* ^1,2,3^	R	R	R	R	R	N/A	R	N/A	S
*P. aeruginosa* ^1,2,3^	R	R	R	S	S	N/A	R	N/A	S
*P. aeruginosa* ^1,2,3^	R	R	R	R	R	N/A	R	N/A	S
*P. aeruginosa* ^1,2,3^	R	R	S	S	R	N/A	R	N/A	S
*E. coli*	R	R	S	R	S	S	R	S	S
*E. coli*	R	R	S	R	R	S	R	S	S
*E. coli*	R	R	S	R	R	S	R	S	S
*K. pneumoniae*	R	R	S	S	R	S	S	S	S
*K. pneumoniae*	R	R	S	S	R	S	R	S	S

^1^ Penicillins: piperacillin and/or piperacillin/tazobactam; ^2^ Cephalosporins: ceftazidime and/or cefepime; ^3^ Carbapenems: ertapenem was excluded for intrinsic resistance. Abbreviations: *P. aeruginosa*, *Pseudomonas aeruginosa*; *E. coli*, *Escherichia coli*; *K. pneumoniae*, *Klebsiella pneumoniae*; R, resistant; S, susceptible; N/A, not applicable for intrinsic resistance.

## Data Availability

The datasets generated during and/or analyzed during the current study are available from the corresponding author on reasonable request.
